# Human Verbal Memory Encoding Is Hierarchically Distributed in a Continuous Processing Stream

**DOI:** 10.1523/ENEURO.0214-18.2018

**Published:** 2019-03-04

**Authors:** Michal T. Kucewicz, Krishnakant Saboo, Brent M. Berry, Vaclav Kremen, Laura R. Miller, Fatemeh Khadjevand, Cory S. Inman, Paul Wanda, Michael R. Sperling, Richard Gorniak, Kathryn A. Davis, Barbara C. Jobst, Bradley Lega, Sameer A. Sheth, Daniel S. Rizzuto, Ravishankar K. Iyer, Michael J. Kahana, Gregory A. Worrell

**Affiliations:** 1 Department of Neurology, Mayo Clinic, Rochester, MN 55905; 2 Multimedia Systems Department, Gdansk University of Technology, Faculty of Electronics, Telecommunications and Informatics, Gdansk, Poland 80233; 3 Department of Physiology and Biomedical Engineering, Mayo Clinic, Rochester, MN 55905; 4 Department of Electrical and Computer Engineering, University of Illinois, Urbana-Champaign, IL 61801; 5 Institute of Informatics, Robotics and Cybernetics, Czech Technical University, Prague, Czech Republic 16000; 6 Department of Neurosurgery, Emory University, Atlanta, GA 30322; 7 Department of Psychology, University of Pennsylvania, Philadelphia, PA 19104; 8 Department of Neurology, Thomas Jefferson University Hospital, Philadelphia, PA 19107; 9 Department of Neurology, University of Pennsylvania Hospital, Philadelphia, PA 19104; 10 Department of Neurology, Dartmouth-Hitchcock Medical Center, Lebanon, NH 03756; 11 Department of Neurosurgery, UT Southwestern Medical Center, Dallas TX 75390; 12 Department of Neurosurgery, Baylor College of Medicine, Houston TX 77030

**Keywords:** cognition, cortical mapping, electrocorticography, high-frequency oscillations, network oscillations

## Abstract

Processing of memory is supported by coordinated activity in a network of sensory, association, and motor brain regions. It remains a major challenge to determine where memory is encoded for later retrieval. Here, we used direct intracranial brain recordings from epilepsy patients performing free recall tasks to determine the temporal pattern and anatomical distribution of verbal memory encoding across the entire human cortex. High γ frequency activity (65–115 Hz) showed consistent power responses during encoding of subsequently recalled and forgotten words on a subset of electrodes localized in 16 distinct cortical areas activated in the tasks. More of the high γ power during word encoding, and less power before and after the word presentation, was characteristic of successful recall and observed across multiple brain regions. Latencies of the induced power changes and this subsequent memory effect (SME) between the recalled and forgotten words followed an anatomical sequence from visual to prefrontal cortical areas. Finally, the magnitude of the memory effect was unexpectedly found to be the largest in selected brain regions both at the top and at the bottom of the processing stream. These included the language processing areas of the prefrontal cortex and the early visual areas at the junction of the occipital and temporal lobes. Our results provide evidence for distributed encoding of verbal memory organized along a hierarchical posterior-to-anterior processing stream.

## Significance Statement

Verbal memory is a complex function supported by a network of brain regions specialized for perception, decision making, and execution of action. Our results shed light on the temporal and anatomic organization of this network during encoding of memories for subsequent recall. By finding consistent differences in fast γ activity recorded directly from the human brain during presentation of words that were later recalled or forgotten, we identified specific regions with the greatest memory effect. This subsequent memory effect was present across a feed-forward processing stream, providing evidence for hierarchical and distributed organization of verbal memory. The identified brain regions in the processing stream present new targets for brain modulation technologies to treat verbal and cognitive deficits in brain disorders.

## Introduction

Are memories encoded in widespread cortical areas or rather in a specialized network of brain regions? In other words, is memory processing distributed or localized in the brain? Our ability to remember specific facts and events from our sensory experiences, defined as declarative memory, is thought to be supported by a medial temporal lobe system (Squire and Zola-Morgan, 1991), comprising the hippocampus and the connected parahippocampal cortical regions. Other regions in the prefrontal and the lateral temporal cortex have also been implicated in the brain network for declarative memory (Eichenbaum, 2000). Another view proposes that memory function is widely distributed across brain areas processing sensory, motor, and higher-order information about the remembered stimuli, including the medial temporal lobe. These multi-modal computations are distributed across multiple cortical areas (Mesulam, 1990; Gaffan, 2002; Rissman and Wagner, 2012) and stored as induced changes in neural activity. In this view, the same areas processing the multi-modal information about a given object are also engaged in encoding its distributed memory trace.

Encoding of words and their multi-modal concepts is arguably one of the most complex tasks relative to other sensory stimuli. Even a simple word like “fish” can be represented and remembered not only in terms of the visual features but also the associated actions of “swimming,” “catching,” or “eating,” as well as other semantic associations with similar animals, names, or symbols (e.g., the ichthys symbol in Christianity). A recent brain imaging study suggests that concepts of words are sparsely encoded and “tile” the entire neocortex in patterns reflecting their semantic modalities (Huth et al., 2016). Declarative memory for verbal information is known to engage both the distributed modality-specific brain areas and those supporting language and other supramodal functions (Binder and Desai, 2011; Wang et al., 2018). It remains unknown, however, if they all contribute to memory encoding of the information about words as well as the objects they describe and, if so, how is it organized in time and anatomic space. Alternatively, it may also be centered in a specialized brain network.

To address these questions, we investigated intracranial recordings taken directly from the human brain in a large number of patients performing a classic paradigm of free recall verbal memory tasks (Kahana, 2012). The tasks probe the declarative memory for words presented for subsequent test of near-immediate free recall. Direct recordings of high-frequency activities (>60 Hz) have been used to study the dynamics of neural processes underlying cognitive functions with superior spatiotemporal resolution (Crone et al., 2006; Jerbi et al., 2009; Lachaux et al., 2012; Johnson and Knight, 2015). They comprise oscillatory and other asynchronous activities (Kucewicz et al., 2017), which are temporally coupled with firing discharges of neuronal populations (Rich and Wallis, 2017; Watson et al., 2017). In the free recall tasks, spectral power of these discharges in the high γ frequencies is different during encoding of subsequently recalled and forgotten words (Sederberg et al., 2007; Long et al., 2014). Less is known about the distribution of this effect in anatomic space and time of stimulus processing. Previous studies quantified the memory effect in selected brain regions (Long et al., 2014) during an early and a late phase of memory encoding (Burke et al., 2014). Therefore, here we employ the subsequent memory effect (SME) in high γ activity as a simple biomarker of the temporal pattern and the magnitude of memory encoding. In contrast to the previous studies describing this biomarker only in a subset of three to seven brain regions (Burke et al., 2014; Kucewicz et al., 2014, 2017; Long et al., 2014), a complete whole-brain picture is provided to elucidate the localization and the spatiotemporal dynamics of verbal memory encoding. Our hypothesis is that verbal memory is encoded across a distributed network of specific brain regions rather than in a localized brain system.

## Materials and Methods

### Study participants

A total of 186 patients undergoing intracranial electroencephalographic monitoring as part of their clinical treatment for drug-resistant epilepsy were recruited to participate in this multi-center collaborative study. Data were collected from the following clinical centers: Mayo Clinic, Thomas Jefferson University Hospital, Hospital of the University of Pennsylvania, Dartmouth-Hitchcock Medical Center, Emory University Hospital, University of Texas Southwestern Medical Center, and Columbia University Hospital. The research protocol was approved by the respective Institutional Review Board at each clinical center, and informed consent was obtained from each participant. Electrophysiological data were collected from standard clinical subdural and penetrating depth electrodes (AdTech Inc., PMT Inc.) implanted on the cortical surface and into the brain parenchyma, respectively. The subdural electrode contacts were arranged either in a grid or a strip configuration with contacts separated by 10 mm. The depth electrode contacts were separated by 5- to 10-mm spacing. In each case, the placement of the electrodes was determined by a clinical team whose sole purpose was to localize seizures for possible epilepsy surgery or implantation of a stimulation device for treatment of seizures.

### Anatomic localization and brain surface mapping

Cortical surface parcellations were generated for each participant from pre-implant MRI scans (volumetric T1-weighted sequences) using Freesurfer software (RRID:SCR_001847). The hippocampus and surrounding cortical regions were delineated separately based on an additional 2-mm-thick coronal T2-weighted scan using the Automatic Segmentation of Hippocampal Subfields (ASHS) multi-atlas segmentation method. Electrode contact coordinates derived from co-registered postimplant CT scans were then mapped to the pre-implant MRI scans to determine their anatomic locations. For subdural strips and grids, the electrode contacts were additionally projected to the cortical surface using an energy minimization algorithm to account for postoperative brain shift. Contact locations were reviewed and confirmed on surfaces and cross-sectional images by a neuroradiologist. The T1-weighted MRI scans were also registered to the MNI152 standard brain to enable comparison of recording sites in a common space across subjects. Anatomic locations of the recording sites, including the Brodmann areas, were derived by converting MNI coordinates to Talairach space and querying the Tailarach daemon (www.talairach.org).

### Electrophysiological recordings

Intracranial data were recorded using one of the following clinical electrophysiological acquisition systems specific to a given site of data collection: Nihon Kohden EEG-1200, Natus XLTek EMU 128, or Grass Aura-LTM64. Depending on the acquisition system and the preference of the clinical team, the signals were sampled at either 500, 1000, or 1600 Hz and were referenced to a common contact placed either intracranially, on the scalp, or on the mastoid process. For analysis, all recordings using higher sampling rates were down-sampled to 500 Hz. A bipolar montage was calculated *post hoc* for each subject by subtracting measured voltage time series on all pairs of spatially adjacent contacts. This resulted in *N* – 1 bipolar signals in case of the penetrating and the strip electrodes, and *N* + x bipolar signals for the grid electrodes, where *N* is the number of electrode contacts and x is the number of extra combinations of bipolar contacts that resulted from the montage.

### Memory tasks

**Figure 1.**
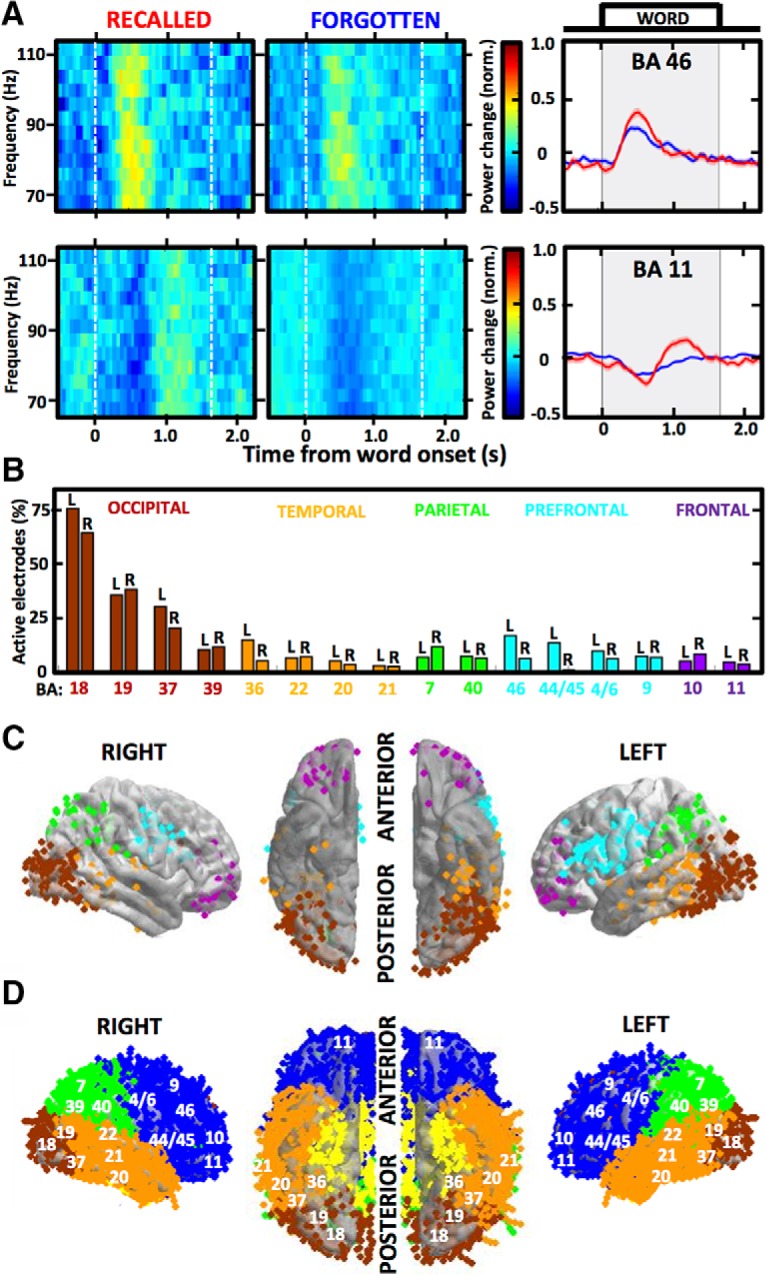
High γ responses to word presentation reveal distributed brain regions activated during memory encoding. ***A***, Spectrograms and mean power plots show trial-averaged high γ responses (aligned to word onset at time 0; shaded area indicates word presentation on the screen) of two example active electrodes localized in Brodmann areas (BAs) 46 (top) and 11 (bottom). Notice the differences between trials with recalled (red) and forgotten (blue) words, defined as the SME. ***B***, Proportions of active electrodes out of all localized in each of the 16 regions identified as activated during memory encoding are color-coded according to the cortical lobe and split between the two hemispheres (L & R label). Notice the highest proportions in the occipital cortical regions and the most consistent hemispheric disparity in the prefrontal cortical regions, especially BA 44/45. ***C***, Average brain surface plots visualize the distribution of all electrodes (each dot is one color-coded electrode contact) pooled from all patients to reveal the activated regions. Notice the differences in hemispheric laterality, especially in the two main clusters of activity aggregated around the occipito-temporal lobe junction and around the ventrolateral prefrontal cortex. ***D***, Brain coverage of all implanted electrodes is presented on the average surface plot as in ***C*** with labels of the studied BAs from ***B***.

The tasks were based on classic paradigms for probing verbal short-term memory (Kahana, 2012), in which subjects learned lists of words for subsequent recall (Fig. 1*A*). Subjects were instructed to study lists of individual words presented sequentially on a laptop computer screen for a later memory test. Lists were composed of 12 words chosen at random and without replacement from a pool of high-frequency nouns (either English or Spanish, depending on the participant’s native language; http://memory.psych.upenn.edu/WordPools). Each session had a set of 25 specific lists using words from the same general pool. The words on each list were either sampled from specific categories like vehicles, music instruments and vegetables, or they were sampled randomly. Each word remained on the screen for 1600 ms, followed by a random jitter of 750- to 1000-ms blank interval between stimuli. Immediately following the final word in each list, participants performed a distractor task (20 s) consisting of a series of arithmetic problems of the form A + B + C = ??, where A, B, and C were randomly chosen integers ranging from 1 to 9. Following the distractor task subjects were given 30 s to verbally recall as many words as possible from the list in any order. Vocal responses were digitally recorded by the laptop computer and later manually scored for analysis. Each session consisted of 25 lists of this encoding-distractor-recall procedure. A total of 165 subjects who remembered >15% of words or completed >12 task lists were included in further analysis. In total, these subjects provided recordings from 24,315 electrodes that were used in this study.

### Electrophysiological analysis

Brain activity induced by word presentation was analyzed in this study, and comprised 1600 ms of word display on the screen and 700-ms blank interval before and after each word (total of 3000-ms epoch). Hence, one complete session yielded electrophysiological signal from 300 word encoding epochs (25 lists × 12 words). The raw signal of each epoch was spectrally decomposed into 50-ms time bins using multi-taper Fast Fourier Transform [Chronux toolbox, RRID:SCR_005547 (Bokil et al., 2010); taper parameters: 4-Hz bandwidth, 250-ms timewidth, 1 taper]. To estimate power in the high γ (65–115 Hz) frequency band, the epoch signal was bandpass filtered between 65 and 115 Hz cutoff frequencies (Bartlett–Hanning, 1000 order) before the spectral decomposition to reduce any possible influence of lower frequencies on the power estimate. The cutoff frequencies for the high γ band were chosen to minimize contamination of the 60-Hz line noise and its first harmonic at 120 Hz. The decomposed spectral power values in a given frequency band were log and *z* score transformed in each frequency bin to account for the power law effect and obtain values that can be compared in the same normative scale (SDs above or below the mean) across sessions and subjects. This *z* score normalization was calculated for each datapoint “i” within any one signal epoch of word presentation according to the following formula:

$$z_{{}_{i}}=\frac{x_{i}-\bar{x}}{s}$$

where X is the raw signal, μ is the mean, and σ is the SD, assuming normal distribution of the sample population. This method is more appropriate than baseline or grand average normalization for signals with non-stationary baseline periods with negative amplitude changes. Normalization within each epoch separately was used to avoid influence of signal non-stationarities across time of a single session or across consecutive sessions. This method, however, is prone to augmentation of any negative or positive power changes from the average estimated within a single epoch. There are alternative options to avoid this potential confound, including normalization across all epochs in a session or normalization to the pre-stimulus baseline.

**Figure 2.**
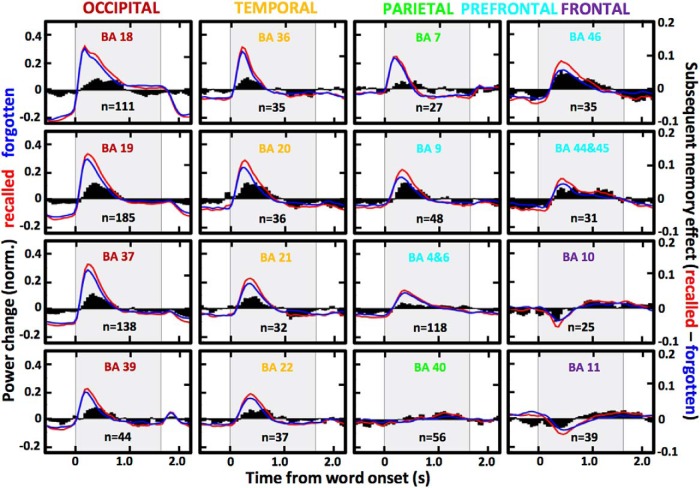
Temporal pattern of the high γ responses and memory effect across all of the activated brain regions. Trial-averaged power changes in high γ activity (as in Fig. 1*A*) are summarized as mean plots for all active electrodes localized in each of the identified brain areas pooled from all patients (*n* indicates the number of electrodes; BA stands for a given Brodmann area region color-coded with respect to the cortical lobe). Black bar plots quantify the SME difference between the two recall conditions (in red and blue) on the right-side *y*-axes. Gray background marks the interval of word presentation. Notice that despite different latencies and amplitudes of the power responses, there is a consistent spatiotemporal pattern of SME magnitude peaking at specific latencies from stimulus presentation across anatomically arranged brain regions, even in case of the late responses observed with the frontal pole electrodes (black bar plots are positive toward the end of word encoding).

**Figure 3.**
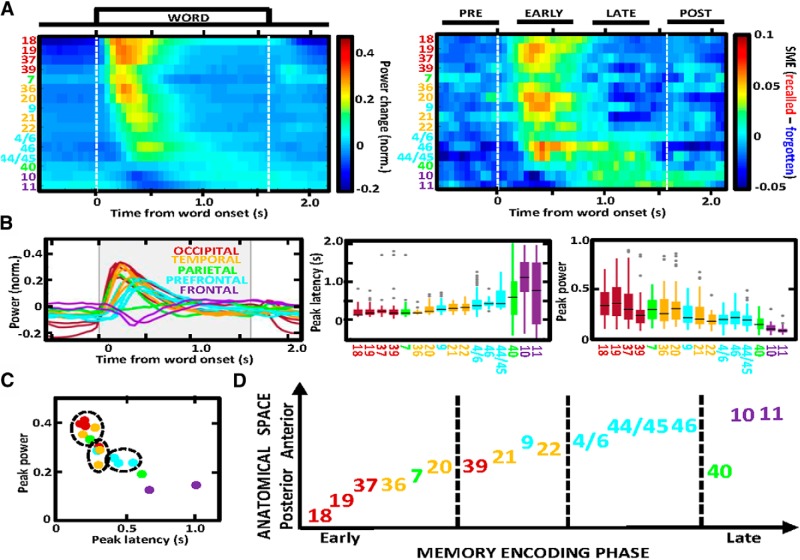
The SME follows a hierarchical sequence of visual information processing. ***A***, Heat map matrices visualize the power and SME plots from Figure 2 across the identified brain areas ordered by their latency of the peak power response. Notice the overlapping order of latencies in power responses (left) and SME (right). ***B***, Summary of all overlaid power responses (left) reveals a temporal sequence of propagation from the occipital to the frontal lobe with gradually decreasing amplitude (left) across the time of word encoding (gray background marks word presentation). Box plots compare latencies and amplitudes (right) at peak of the power response across the sequence of brain regions. Notice the consistent trend of increasing latency and decreasing amplitude along the stream. ***C***, Scatterplot reveals a correlation between the latency and amplitude of the high γ response. Clusters of the identified brain regions (each dot is one color-coded region) form groups (dash-line circles) based on hierarchical clustering of the mean latency and peak power estimates from ***B***. ***D***, Flowchart of the hypothetical processing stream for verbal memory encoding, following a proposed anatomic and temporal feed-forward order. Dashed lines separate distinct phases of memory encoding based on the clustering in ***C***. Notice hierarchical organization of the stream starting in the early visual areas and culminating in the higher-order prefrontal cortical areas.

Trial-averaged power estimates of high γ activity were calculated for every electrode using all epochs with words that were subsequently recalled or forgotten. Electrodes that were “active” during word encoding were selected based on consistent power changes quantified as SD of the trial-averaged estimate >0.05 (as in Fig. 1*A*). Electrodes with the SD of their trial-averaged estimate <0.05 were considered “not active” during word encoding and were excluded from further analysis. The analysis focused on the majority of active electrodes, which showed increased high γ power in response to word presentation, as opposed to the remaining electrodes with decreased power or a mixed response. This electrode selection of the automatically identified active electrodes was manually performed based on visual inspection of the profile of trial-averaged power change (examples in Fig. 1*A*). Proportion of active electrodes was determined using the overall number from all subjects and the total number of electrodes localized in a particular brain area (Fig. 1*B*). We set a conservative threshold of 25 active electrodes from at least 10 different subjects for a given brain area to be included in the analysis and calculation of the grand average power change plots from all active electrodes localized in a given Brodmann area (Fig. 2). Due to a small number of electrodes implanted in any one Brodmann area of a single patient, the active electrodes from specific Brodmann areas were pooled from all patients into pseudo-populations to compare brain responses in the identified brain regions. SME was calculated by subtracting the grand-average power estimate from the recalled and the forgotten word conditions in each of the 50-ms bins (Fig. 2). Brain regions were ordered in sequence in increasing order of the latency of peak power response in the grand-average power plots (Fig. 3*A*). Peak power and latency values were compared using the bin showing the maximum power in the trial-averaged power plot of each electrode (Fig. 3*B*). Mean SME values were obtained by taking the mean amplitude in four segments of the encoding epoch (Fig. 3*A*): pre-encoding (–500–0 ms), early encoding (200–700 ms), late encoding (900–1400 ms), and post-encoding (1600–2100 ms) relative to the onset or word presentation.

### Statistics

All statistical tests were performed in MATLAB (MathWorks Inc., RRID:SCR_001622) using built-in and custom written codes. Box plots were used to compare the medians, interquartile interval, range and outliers of data point distributions for the latency and power at peak maximum of the high γ response to word presentations (Fig. 3*B*). We used hierarchical clustering to group the identified active brain areas (Fig. 3*C*) by the mean estimates of peak power and latency, as presented in Figure 3*B*, right. The mean values were evaluated by the clustering algorithm to determine subgroups of highest similarity, which were then used to group the regions involved in the early and late phases of memory encoding (Fig. 3*C*,*D*). One-way ANOVA test compared mean SME values across the identified active brain areas in the four segments of the encoding phase. *Post hoc* Tukey–Kramer test was used to compare 95% C.I. of the mean across the identified regions, corrected for multiple comparisons (MATLAB, MathWorks Inc.). Brain regions with the greatest SME magnitude were determined with descriptive statistics by determining the upper quartile of the absolute SME values, including both positive and negative SME. Data are shown as mean ± SEM.

## Results

In total, we analyzed intracranial recordings from 24,315 bipolar electrodes implanted in 165 patients, who performed the same free recall verbal memory tasks. This provided coverage of almost the entire cortical surface and subcortical structures (Fig. 1), including the amygdala and the hippocampus. We identified 1665 of these electrodes (6.85%) that were defined as active during memory encoding by showing consistent high γ activity responses to presentation of words to be remembered for subsequent recall (Fig. 1*A*). Most of these active electrodes showed a pattern of increased high γ power following the presentation, which was preceded by a suppression of power in particular brain regions, as exemplified by the two selected electrodes in Figure 1. To obtain robust patterns of the high γ responses, we identified 16 Brodmann area regions that showed consistent active electrode responses in multiple electrodes pooled from all patients into pseudo-populations that were used in all subsequent analyses (Fig. 1*B*,*C*). Apart from the 16 identified brain regions, only a small number of the active electrodes from a few patients were found in the primary visual cortex (*n* = 11), somatosensory cortex (*n* = 15), posterior cingulate cortex (*n* = 22), auditory cortex (*n* = 12), hippocampus (*n* = 5), and amygdala (*n* = 1), among others, as measured with the induced high γ activity. The highest proportion of the active electrodes was observed in the visual processing areas of the occipital cortex, reaching 75% of all implanted electrodes (Fig. 1*B*), as compared to the other activated areas showing proportions below 20%. There were no consistent differences between hemispheres, except for the four prefrontal cortical areas, which all had higher proportions in the left hemisphere. Most of these prefrontal active electrodes were localized in proximity to the Broca’s speech area (Brodmann area 44 and 45 in the language-dominant hemisphere) where this hemispheric disparity was the largest (Fig. 2*B*,*C*). This prefrontal cortical region comprised one of the two main clusters of active electrode density together with areas around the occipito-temporal lobe junction (Fig. 1*C*). The selective clustering of active electrodes was not related to denser sampling of implantation in these regions (Fig. 1*D*) compared to others and is congruent with the semantic brain network for processing verbal information (Binder et al., 2009; Riès et al., 2017).

The two example electrodes from Figure 1 demonstrate differences in the high γ power response between trials with words that were subsequently recalled and those that were forgotten, here defined as the SME. We summarized these differences for all active electrodes pooled from each of the identified Brodmann area regions and found common temporal patterns of SME dynamics in all of the brain regions. The pooled electrode populations showed positive SME (i.e., more high γ power on the recalled word trials) peaking at specific phases of word encoding according to the anatomic location (Fig. 2). The memory effect was present in all brain regions, despite specific differences in the profile of SME latency and magnitude. Occipital cortex regions showed the shortest latencies and the highest magnitudes of the high γ power induced by word presentation relative to the other more anterior regions with gradually longer latencies and decreased amplitude of the power response. All brain regions from the early visual processing areas in the occipital lobe through to higher-order association areas in the frontal lobe showed the memory effect with region-specific differences in magnitude.

In contrast to the power response, SME magnitude did not show a gradual decrease from the early visual to the late processing areas. The greatest memory-related differences between the trials with recalled and forgotten words were found in specific brain regions at various times (Fig. 2). To explore this heterogeneity of the greatest SME localization we arranged the 16 brain regions according to the latency of their peak power response (Fig. 3). High γ responses revealed a sequential stream of induced power smoothly propagating from the most posterior visual Brodmann area 18 in the occipital lobe continually to the most anterior areas 10 and 11 in the frontal pole (Fig. 3*A*). The amplitude of these responses was gradually decreasing along the propagation stream and had the lowest values with the poorest estimates of the peak latency in the last three brain regions of Brodmann areas 40, 10, and 11 (Fig. 3*B*), where inconsistent peaks occurring at different latencies were observed. Surprisingly, latencies of the SME followed the same sequence of propagation. SME amplitudes, in contrast, did not show the same gradual decrease in magnitude as found in the power response, but instead revealed the highest values in clusters of specific brain regions both at the top and at the bottom of the stream (Fig. 3*A*). We noticed that the greatest amplitude of the memory effect followed the peak power response in time. In general, we found a consistent pattern of gradually increasing latency and decreasing amplitude of the high γ response along the processing stream (Fig. 3*B*). The two variables showed a correlation across the identified brain regions (Fig. 3*C*). Given the temporal organization of the high γ responses, we grouped the activated brain regions into clusters of similar peak latency and power values. There were four major subgroups separating the activated regions into the early, intermediate and late phases of memory encoding. The temporal sequence of the groups correlated with continuous posterior-to-anterior anatomic progression of information processing (Fig. 3*D*).

**Figure 4.**
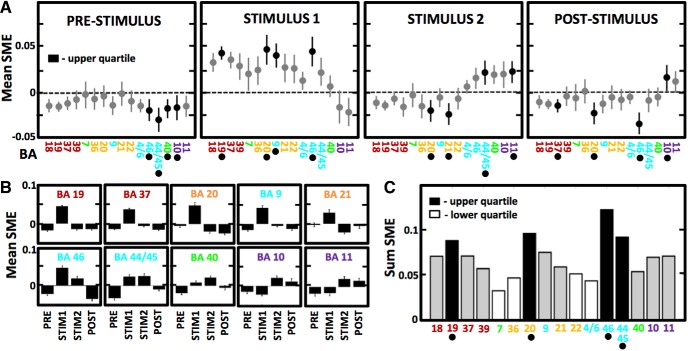
Distributed prefrontal and occipito-temporal lobe regions of the semantic brain network show the greatest memory effect. ***A***, ANOVA comparison of mean SME amplitude in 500-ms segments before and after word presentation (as indicated in Fig. 3*A*) showed a significant effect of brain region in all four phases of memory encoding (*p* < 0.01, *F* > 2.0), displayed as Tukey–Kramer *post hoc* comparison of the means and error bars (95% C.I.) corrected for multiple comparisons. Regions in the upper quartile of the highest absolute magnitude are marked in black and indicated by dots below the *x*-axis labels. Notice the greatest magnitude in the EARLY phase immediately following word presentation, and positive SME in the last two phases confined to the higher-order areas of the processing stream. ***B***, Summary of the mean SME values across the four phases is displayed for ten areas of the upper quartile in ***A***. ***C***, Grand summary of absolute total SME magnitude identifies four regions with the greatest (marked in black and by dots below the *x*-axis labels) and the lowest (marked in white) memory effect. Notice a widespread distribution of SME, which is the highest in Brodmann areas 44/45, 46, 19, and 20 associated with visual and semantic information processing.

Finally, we asked where in the processing stream is memory effect the greatest? We addressed this question by comparing mean SME magnitude across the activated brain regions in four segments of memory encoding (Fig. 4*A*). The segments were selected to capture distinct phases of stimulus processing: preparation before word presentation (PRE), early and late processing of the presented words (EARLY, LATE) and any processing after the presentation (POST). We found a significant effect of the region on SME magnitude in all phases (ANOVA, 15 d.f.; PRE: *F* = 2.14, *p* = 0.0069, EARLY: *F* = 13.31, *p* < 0.0001, LATE: *F* = 13.01, *p* < 0.0001, POST: *F* = 5.74, *p* < 0.0001). Although we found a different set of regions showing the highest absolute SME magnitude (positive or negative; upper quartile) in each phase (Tukey–Kramer *post hoc* comparison of the means), a subset of them was repeatedly found in at least two of the four phases (Fig. 4*A*). Each one of the regions showed a specific profile of SME magnitude across the four phases of memory encoding (Fig. 4*B*). In general, regions in the beginning of the processing stream had positive SME only in the EARLY phase, whereas regions in the end of the stream had positive SME also in the LATE and POST phases. The greatest total SME magnitude, which was determined by summing the absolute mean values from the four phases, was localized to Brodmann areas 44/45 and 46 in the ventrolateral prefrontal cortex, and Brodmann areas 19 and 20 in the occipito-temporal lobe junction (Fig. 4*C*). These areas overlap with brain regions showing high density of active electrodes (Fig. 1*C*), which have been associated with speech (Flinker et al., 2015) and with visual processing (Mano et al., 2013) of semantic information, respectively. This finding does not mean that the memory effect was present there only, but rather that it was relatively greater compared to all other brain regions.

## Discussion

Our results suggest that verbal memory is encoded in a hierarchical sequence corresponding to the anatomic stream for information processing. Classic experiments in the visual cortex (Wurtz, 2009) introduced a hypothesis that selective neuronal responses to stimuli of increasing complexity are localized in successive order of cortical areas. Simple stimulus features like points and edges processed in the early sensory areas would feed-forward their outputs to consecutive areas to combine basic features into higher-order visual information about forms and shapes (Hubel and Wiesel, 1962). These outputs, in turn, would eventually feed on to computations of complex objects like faces recorded in associative areas of the temporal cortex, forming a hierarchical sequence (Riesenhuber and Poggio, 1999). Two major visual processing streams have been proposed for processing of objects and actions (Ungerleider and Mishkin, 1982; Desimone and Ungerleider, 1989), originating from the primary sensory occipital cortex and diverging into the temporal and parietal cortical areas through to the prefrontal cortex. Experimental evidence for the processing streams has thus far been limited to experiments using focal lesions, recordings, and modeling in specific cortical systems, and more recently to brain imaging studies (Milner and Goodale, 2006).

In our study, we took advantage of direct brain recordings from a large number of patients to track the hypothetical sequence of responses to the presented word stimuli throughout the cortex. Previous studies with fewer patients showed a temporal progression in high-frequency responses from occipital to the prefrontal cortical lobe (Kucewicz et al., 2014) with a clear distinction between an early and a late phase observed in selected cortical gyri (Burke et al., 2014). Analogous progression was also reported in the temporal and prefrontal cortical areas in response to auditory stimuli (Canolty et al., 2007). Here, we were able to track a continuous sequence of the induced high γ activity and, for the first time, of the SME on the level of specific Brodmann areas across the entire cortical surface (Fig. 1). Due to a smaller number of patients and electrodes available, previous studies were limited to analyzing high γ activity only at the level of selected cortical lobes (Kucewicz et al., 2014), gyri and brain structures (Burke et al., 2014), or a range of neighboring Brodmann areas (Kucewicz et al., 2017). Having the advantage of a complete coverage of the cortical surface (Fig. 1*D*), we were able to quantify high γ electrophysiological activity from all Brodmann areas. Relative latencies of this activity and SME revealed a sequential continuous order congruent with the anatomic and functional organization of the brain, starting in the early sensory areas of the visual cortex and progressing through the associative areas of the temporal, parietal, and frontal cortex. It is important to note that the biomarker of high γ responses was averaged over multiple trials and electrodes from different patients, which would explain the “blurring” of the response across time in Figure 3. The response was relatively sharp and confined in time for the areas early in the processing stream, in comparison with smoother responses in the late areas, which were more extended in time. One would expect these later associative areas supporting higher-order processes to be more variable in terms of their activation time compared to a more stereotypical pattern in the early processing areas. Precise timing in this sequence of progression could still be further resolved with local recordings of single unit and field potential activity. Most recent study in non-human primates confirmed the hierarchical organization and analogous spatiotemporal progression of neuronal spiking activity during short-term memory processing (Dotson et al., 2018). Another recent study showed a close relationship between neuronal spiking and field potential activity in the high γ band (Rich and Wallis, 2017), concluding that the high γ activity is a useful biomarker of large-scale information processing. Our results with ECoG recordings of high γ activity corroborate current evidence from the brain imaging studies for the visual processing stream (Milner and Goodale, 2006) and now provide supreme spatiotemporal resolution of the high γ biomarker and the newly mapped memory effect to study the underlying neurophysiology.

Both the brain imaging studies and the intracranial recordings that we employed in this study probe common neurophysiological processes. Spectral power of the high-frequency activity has been shown to correlate with the BOLD signal detected in the imaging studies (Logothetis et al., 2001; Niessing et al., 2005) and proposed to reflect general activation of neuronal populations. High γ activity, therefore, offers an intermediate biomarker of localized neuronal firing to bridge the gap between the non-invasive imaging techniques and the invasive single neuron recordings during cognitive functions (Crone et al., 2006; Jerbi et al., 2009; Lachaux et al., 2012). For example, this biomarker can be used to effectively map language areas in the brain before surgical treatment instead of direct electrical stimulation to acutely disrupt language functions (Wang et al., 2016). The same goal can now be achieved by non-invasively mapping the biomarker activity with magnetoencephalography during a simple reading task (Dalal et al., 2009). Here, we used the high γ biomarker not only to map the areas activated during word encoding, but also to quantify their contribution to memory as SME. Predicting subsequent recall with this biomarker (Sederberg et al., 2007; Long et al., 2014) and with the BOLD signal (Kim, 2011) has proven fruitful for investigating memory and cognition, but its dynamics has not been explored. The high γ activity presents unique advantages for studying the physiologic mechanisms and the spatiotemporal dynamics of memory processing. Compared to the BOLD signal used in functional brain imaging, it offers improved temporal resolution and thus a mechanistic insight into the role of brain oscillations in memory, despite a relatively lower spatial resolution. This mechanistic insight is also possible with scalp EEG signals but these have considerably lower spatial resolution of the field potential compared with the intracranial electrodes sampling the local high γ activity. Still, when used as a biomarker of the subsequent recall it may also reflect other associated processes like attention, perception or decision making required for successful memory encoding.

All of the activated cortical areas showed SME, i.e., differences in the high γ response between the subsequently recalled and forgotten words. The differences revealed consistent temporal pattern across consecutive areas of the visual processing stream, in which peak of the memory effect occurred at gradually longer latencies from word presentation. Temporal profile of SME was previously only studied and reported in the occipital cortex responses in these tasks (Kucewicz et al., 2017). It was not quantified in the temporal or magnitude context of all other brain regions of the processing stream that were previously reported to show SME (Burke et al., 2014; Kucewicz et al., 2014, 2017; Long et al., 2014). This subsequent effect of memory encoding was not expected to be observed most strongly in a subset of brain regions in the top and in the bottom of the processing stream among the other activated brain areas. Figure 3 shows the same temporal progression of the SME peak following the peak of the induced high γ power in consecutive areas of the processing stream. Our results suggest that a widespread network of areas processing the visual and semantic information is involved to various degree in encoding memory for the words as quantified with the SME magnitude. This conclusion is supported by evidence from a recent study of the high γ responses in word retrieval (Riès et al., 2017), which argues against the modular view of a localized area for a particular semantic function. Instead, the authors proposed a widespread network of areas for general lexical-semantic processing with overlapping nodes in the left prefrontal and the occipito-temporal cortex.

These two brain regions had the largest density of active electrodes and relatively high SME in our tasks. They are both implicated with the semantic network for processing language (Binder et al., 2009; Binder and Desai, 2011; Wang et al., 2018). The semantic network is thought to be widely distributed and comprise several sub-networks processing modal information about visual, phonological or verbal features, and the supra-modal linguistic information. High γ activity was used to identify these different sub-networks (Vidal et al., 2012; Collard et al., 2016), providing a useful signal for analyzing the dynamics of information processing across distributed semantic networks. The two regions in the prefrontal and the occipito-temporal cortex constitute critical nodes in these networks. We found the greatest SME magnitude in the Brodmann areas within these regions, which included the Broca’s area historically associated with speech production. The actual role of Broca’s area in the semantic network may, however, be more general in light of the recent evidence from another study of the intracranial high γ activity (Flinker et al., 2015). The authors proposed that it “coordinates transformation of information across large-scale cortical networks involved in word production.” The role performed by these prefrontal areas, together with the areas in the occipito-temporal cortex (Mano et al., 2013), would be critical for successful encoding of memory for words and thus explain the highest magnitude of the SME found in these two brain regions. The identified Brodmann areas 19, 20, as well as area 37, which was also ranked high in the total SME score (Fig. 4*C*), are involved in processing both the patterns of letters and words (BA19 and BA37) and more complex information about objects described by the words we used in the tasks (BA20). Our findings suggest that both types of computation were engaged and played a role in subsequent memory recall. Importantly, these were not the only regions expressing the memory effect, although they expressed relatively greater SME magnitudes.

Other measures of memory processing need to be investigated to confirm our findings with ECoG recordings of high γ activity. A SME was also reported in the lower frequency bands (Burke et al., 2014; Long et al., 2014) known to be important for memory and cognitive functions (Siegel et al., 2012). Theta rhythm in the medial temporal lobe, for instance, is another plausible biomarker of memory processing that has not been explored in our study, which may explain the lack of activation in the hippocampus and the associated cortical regions as measured with high γ activity. Phase and amplitude interactions between the low and high-frequency activities presents yet another biomarker to be explored in the future studies. In addition, our study was limited to one behavioral paradigm for verbal memory encoding using short delays (approximately 20–30 s) and minimal contextual information (words were recalled in any order with no relevance to the sequence of presentation). Other paradigms specifically probing the episodic component of verbal memory would be expected to induce greater activation in the medial temporal lobe. Nonetheless, the high γ biomarker identified distinct cortical areas activated in the verbal memory tasks that we employed.

Thus, the areas classically associated with visual processing and speech production are here implicated with successful encoding of declarative verbal memory. Within the limitations of our study methods, we did not find comparable high γ responses in the hippocampus and the associated neocortex, or in the semantic areas of the anterior and medial temporal cortex. The high γ responses and SME were distributed across a widespread network supporting the processes essential to verbal memory (Mesulam, 1990). Our findings are congruent with a non-modular view, in which memory traces are stored across a network of areas processing specific multi-modal representations (Gaffan, 2002; Bussey and Saksida, 2007). In this view, widespread assemblies of neurons communicate encoded information across the network through means of synchronous interactions (Singer, 1993; Varela et al., 2001; Siegel et al., 2012) without a need for one localized memory module in the brain (Knight, 2007). It is important to note that we have only tested short-term memory recall of the encoded information in this study without testing the intermediate or long-term memory encoding. Therefore, the role of the medial temporal lobe system as a critical node in encoding long-term memory representations in our tasks remains to be further explored. There may still be other localized systems of critical nodes. The identified areas in the prefrontal and the occipito-temporal cortex can be tested for their potential roles as the nodes for verbal memory encoding in experiments using focal brain modulation techniques. Direct electrical stimulation for memory enhancement (Kucewicz et al., 2018a,b) would provide a compelling evidence for this ascribed role and yield new targets for therapeutic interventions to treat cognitive deficits.

## Synthesis

Reviewing Editor: Morgan Barense, University of Toronto Decisions are customarily a result of the Reviewing Editor and the peer reviewers coming together and discussing their recommendations until a consensus is reached. When revisions are invited, a fact-based synthesis statement explaining their decision and outlining what is needed to prepare a revision will be listed below. The following reviewer(s) agreed to reveal their identity: Christopher Honey.

We agreed that the findings are interesting and important, and that the revised manuscript was substantially improved. However, we have some remaining concerns. The most substantive is with regard to the z-scoring. In particular, if the analysis included z-scoring of individual epochs of data, then this would explain the "flip over" SME effect, because it would mean that higher activation at one moment in a trial must necessarily be offset by lower activation at another part of the trial. As this is a critical element of the results, it should be addressed. Additional concerns regarding framing and statistical context are described below from each reviewer.

Reviewer 1:

In their revision, the authors have improved the manuscript by measuring single-subject response latencies. I still have some concerns about the interpretation of the subsequent memory effects that appear throughout the cortical hierarchy. I also have a follow-up question about the z-score normalization method, which may introduce a serious artifact, depending on how it was performed.

[Subsequent Memory Effects] In my initial review, I questioned why the authors were emphasizing the presence of a subsequent memory effect (SME) throughout the processing stream. For example, line 313 in the Discussion: "A surprising finding of this study is that all of the activated cortical areas showed SME - all showed differences in the high gamma response between the subsequently recalled and forgotten words. "

I will simply repeat what I wrote in my initial review:

The fact that a region expresses an SME does not require that it is causally involved in supporting the

memory. If I present 20 pictures of blank screens and 20 pictures of objects, then people will recall

the object and will not recall the blank screens. This will produce an SME that is distributed

throughout the visual processing pathway. So it seems that an SME across the entire stream of

activated brain areas is not a surprising result.

The authors responded by noting:

"We agree with the reviewer that a weak response in the early stages of the processing stream would be expected to be followed by proportionally weaker responses in the subsequent stages (as would perhaps be observed with presentation of non-sense words or possibly even a blank screen). In line with this assumption, the induced gamma power revealed gradually decreasing amplitude along the stream (Fig.3a left). "

As far as I can tell, this observation about the gradations in the magnitude of the mean response is not relevant. The mean response (and how it varies across regions) is orthogonal to the question about the SME - the central concern (the SME) depends on the trial to trial variation in response, not the mean. Specifically, the question is whether a larger response on trial N in early areas is associated with larger responses on trial N in other, downstream areas - a "feedforward cascade" which would explain why SMEs are observed throughout all responsive areas.

Later in their response letter the authors write: "Nevertheless, we agree with the reviewer that the unexpected finding is not that the SME is present throughout the entire stream but rather that its magnitude is distributed non-uniformly along the stream in contrast to the induced power response."

If the authors agree that the widespread SME is not surprising, then I cannot understand why the manuscript has a paragraph in the Discussion, line 313, starting with the opposite statement,: "A surprising finding of this study is that all of the activated cortical areas showed SME - all showed differences in the high gamma response between the subsequently recalled and forgotten words. "

[z-score and Flip-Flop] I was unable to tell from the revised text whether

(i) the z-score normalization was applied _separately_ for each 3000ms epoch (i.e. whether the mean and standard deviation were computed separately for each epoch, and then used to normalize that individual epoch)

or

(ii) a common mean and standard deviation were extracted from long, concatenated data across all epochs in a session, and then these common mean and s.d. were used to z-score all epochs at once.

If the z-score normalization was computed separately in each epoch (as in (i)), then there is a problem, because z-scoring each epoch separately could explain the "flip-over" in the SME patterns as a signal-pressing artifact. Specifically, a larger increase during the "active" period of a trial will necessarily produce more negative values during the "non-active" periods of the trial, since the active and non-active periods must sum to zero on each trial. This would call into question the meaningfulness of the flip-over phenomenon - instead it could arise from a subset of the time points in each epoch being above the mean, pushing the remaining points below the mean. Please explicitly state whether the z-scoring parameters were extracted from long stretches of data or from single trial epochs. More generally, please clarify for the reader why the z-score normalization cannot account for the flip-over SME effects that are reported.

[Brodmann Areas] Please consider adding a map of the Brodmann area labels you are using - perhaps to Figure 1 C/D in Figure 3 - so that the reader can more easily understand which anatomical areas you are referencing with numerical Brodmann Area labels in Figure 3, and compare these against the coverage maps shown in Figure 1.

Reviewer 2:

The authors provide a detailed point-by-point response and address all concerns. They acknowledge several shortcomings that arise when such a homogeneous data set is analyzed.

1. how did the authors deal with electrodes that had multiple peaks?

2. boxplots and summary plots should be more informative: Please show individual data points, medians, whiskers or raincloud plots. Anything that shows the actual distribution and not just mean and SEM would help. I think this is crucial for the latency analysis, but applies to all other plots, too.

3. The authors might want to cite Rich and Wallis (2017) Nat Comm and Watson (2017) EJN as resources that link HFA to spiking activity.

4. It should be clearly noted when the author pooled data into a 'pseudo-population'. I think some analysis choices are rather unusual and subjective.

## Author Response

Reviewer 1:

In their revision, the authors have improved the manuscript by measuring single-subject response latencies. I still have some concerns about the interpretation of the subsequent memory effects that appear throughout the cortical hierarchy. I also have a follow-up question about the z-score normalization method, which may introduce a serious artifact, depending on how it was performed.

[Subsequent Memory Effects] In my initial review, I questioned why the authors were emphasizing the presence of a subsequent memory effect (SME) throughout the processing stream. For example, line 313 in the Discussion: "A surprising finding of this study is that all of the activated cortical areas showed SME - all showed differences in the high gamma response between the subsequently recalled and forgotten words. "

I will simply repeat what I wrote in my initial review:

The fact that a region expresses an SME does not require that it is causally involved in supporting the

memory. If I present 20 pictures of blank screens and 20 pictures of objects, then people will recall

the object and will not recall the blank screens. This will produce an SME that is distributed

throughout the visual processing pathway. So it seems that an SME across the entire stream of

activated brain areas is not a surprising result.

The authors responded by noting:

"We agree with the reviewer that a weak response in the early stages of the processing stream would be expected to be followed by proportionally weaker responses in the subsequent stages (as would perhaps be observed with presentation of non-sense words or possibly even a blank screen). In line with this assumption, the induced gamma power revealed gradually decreasing amplitude along the stream (Fig.3a left). "

As far as I can tell, this observation about the gradations in the magnitude of the mean response is not relevant. The mean response (and how it varies across regions) is orthogonal to the question about the SME - the central concern (the SME) depends on the trial to trial variation in response, not the mean. Specifically, the question is whether a larger response on trial N in early areas is associated with larger responses on trial N in other, downstream areas - a "feedforward cascade" which would explain why SMEs are observed throughout all responsive areas.

Later in their response letter the authors write: "Nevertheless, we agree with the reviewer that the unexpected finding is not that the SME is present throughout the entire stream but rather that its magnitude is distributed non-uniformly along the stream in contrast to the induced power response."

If the authors agree that the widespread SME is not surprising, then I cannot understand why the manuscript has a paragraph in the Discussion, line 313, starting with the opposite statement,: "A surprising finding of this study is that all of the activated cortical areas showed SME - all showed differences in the high gamma response between the subsequently recalled and forgotten words. "

This sentence was meant to be changed in the revised manuscript but we missed it. We can now better understand the Reviewer's point and agree that this observation is not critical for the main goal of this study, which was to compare the magnitude of the memory effect across the activated brain regions. We have now revised this first sentence in the fifth paragraph of Discussion as follows:

All of the activated cortical areas showed SME, i.e. differences in the high gamma response between the subsequently recalled and forgotten words.

Later in the paragraph we also corrected the following statement and the first sentence of the next paragraph to shift the emphasis away from the fact that all activated brain areas showed some SME and to focus it on the observation that the SME magnitude was varied across widespread brain regions involved in the semantic network:

Our results suggest that a widespread network of areas processing the visual and semantic information is involved to various degree in encoding memory for the words as quantified by the SME magnitude.

We are grateful for spotting this confusing statement opening the whole paragraph and apologize for failing to correct this sentence in our first revision.

[z-score and Flip-Flop] I was unable to tell from the revised text whether

(i) the z-score normalization was applied _separately_ for each 3000ms epoch (i.e. whether the mean and standard deviation were computed separately for each epoch, and then used to normalize that individual epoch) or

(ii) a common mean and standard deviation were extracted from long, concatenated data across all epochs in a session, and then these common mean and s.d. were used to z-score all epochs at once.

If the z-score normalization was computed separately in each epoch (as in (i)), then there is a problem, because z-scoring each epoch separately could explain the "flip-over" in the SME patterns as a signal-pressing artifact. Specifically, a larger increase during the "active" period of a trial will necessarily produce more negative values during the "non-active" periods of the trial, since the active and non-active periods must sum to zero on each trial. This would call into question the meaningfulness of the flip-over phenomenon - instead it could arise from a subset of the time points in each epoch being above the mean, pushing the remaining points below the mean. Please explicitly state whether the z-scoring parameters were extracted from long stretches of data or from single trial epochs. More generally, please clarify for the reader why the z-score normalization cannot account for the flip-over SME effects that are reported.

Scenario (i) was used to normalize the signal separately within each epoch, in contrast to calculating the z-score across all epochs in a given session - scenario (ii). The problem with (ii) is that we observe large difference in voltage amplitude of the signal sampled from the same electrode between sessions conducted on subsequent days. This variation is probably caused by slight changes in the biophysical environment and/or movement of the electrodes, which would affect the recorded field potential. Such non-stationarities in the recorded signal and other neural adaptation processes (from repeated presentation of the stimuli) could also be observed within a single session, which typically lasted from 40 minutes to over one hour due to breaks in performing the tasks taken by the patients. Hence, amplitude values of the signal sampled from a given electrode in the beginning of a session could be different from the values in the end of the session. In contrast, timescale of a single epoch is too short for such signal non-stationarities to affect the normalization in case of our choice method (i).

Still, we agree that the concern of obtaining artificially negative power changes and SME due to normalization within epoch requires to be checked. Therefore, we compared the power changes and the SME as presented in Figure 2 in an example electrode (top panel) and in a group of electrodes from the same brain region (bottom panel) using three different normalization procedures (Figure R1). The first two ('epoch' and 'session') correspond to scenarios 'i' and 'ii', respectively, as described by the Reviewer, where the (i) z-score transformation was applied separately in each epoch or (ii) taking the signal from all epochs in one session to calculate the mean and standard deviation. The last normalization procedure divided all values by a mean baseline estimate taken from -0.5 to -0.1 s before stimulus presentation ('pre-stimulus') as typically practiced in event-related response analysis. The negative polarity of the power changes and SME in the pre-stimulus and the post-stimulus period was preserved in all three normalization procedures. The magnitude of this negative change was expectedly the lowest for the 'pre-stimulus' normalization. Differences between the 'epoch' and 'session' normalization were relatively small. Given that the normalization suggested by the reviewer does not seem to alter the results and their interpretation, we would prefer to remain with the with-in epoch 'i' normalization to avoid any possible influence of the signal non-stationarities.

Figure R1. Comparison of the effect of three normalization methods on polarity of the induced high gamma power and the subsequent memory effect (SME). Power changes (red and blue line plots, left y-axes) and SME (black bar plots, right y-axes) are plotted as in Fig. 2 for an example active electrode (top row) and for all active electrodes from BA 19 in the same subject (bottom row). Z-score normalization was applied within single epochs ('epoch'), across all epochs in a given session ('session') or without the z-score transformation by dividing by mean baseline estimate ('pre-stimulus') as typically practiced in other studies in the field. Notice that different types of normalization do not affect the negative polarity of power change and SME in the pre- and post-stimulus periods, even though magnitude of these responses is expectedly the smallest for the 'pre-stimulus' normalization. Notice close similarity of responses in case of the 'epoch' and 'session' normalization.

We agree, however, that it is required to clarify the normalization procedure in the manuscript. The fifth paragraph of Methods has now been updated in the following sentence:

This z-score normalization was calculated for each datapoint 'i' within any one signal epoch of word presentation according to the following formula:

And two new sentences were added at the end of the paragraph:

Normalization within each epoch separately was used to avoid influence of signal non-stationarities across time of a single session or across consecutive sessions. Comparison with normalization across all epochs in a session and with normalization to the pre-stimulus baseline was made to control for any possible artifacts of the selected procedure.

[Brodmann Areas] Please consider adding a map of the Brodmann area labels you are using - perhaps to Figure 1 C/D in Figure 3 - so that the reader can more easily understand which anatomical areas you are referencing with numerical Brodmann Area labels in Figure 3, and compare these against the coverage maps shown in Figure 1.

This is an excellent suggestion - we have now added the labels of the studied Brodmann areas to the Figure 1d maps of all electrodes used in the study and updated the figure legend accordingly.

Thanks to this suggestion we have now also spotted a mistake in the Figure 1d brain maps. The medial hemispheric views were incorrectly showing all electrodes from the lateral view. Since there were hardly any electrodes implanted and localized on the medial side of each hemisphere, i.e. in the inter-hemispheric area, it is redundant to show this additional view. Hence, we decided to remove it from the 'c' and 'd' suplot maps. In effect, the clarity of the figure is now improved, overall size is more compact, and the localization of the Brodmann areas is much easier to read. We remain indebted to the Reviewer for the careful reviews which have significantly improved this manuscript.

-

Reviewer 2:

The authors provide a detailed point-by-point response and address all concerns. They acknowledge several shortcomings that arise when such a homogeneous data set is analyzed.

1. how did the authors deal with electrodes that had multiple peaks?

The answer to this comment is provided by the distribution of the peak power and latency values, presented with more detail in our response to the following comment below (Figure R2). In general, we observed that the higher the peak amplitude, the lower and more accurate (less variable) the estimate of the peak response latency, as presented in Figure 3b subplot. In the early stages of our proposed information processing pathway, starting in the visual areas of the occipital cortex through to the prefrontal cortical regions, the peaks of the induced high gamma response had relatively high amplitudes occurring at specific latencies from the time of word presentation. Variation in the latency was relatively small with a narrow interquartile range and few outliers (see Figure R2 below in response to the following comment). This shows that in these regions we typically observed a single distinct peak occurring at a more-or-less constant latency. We only had a problem of multiple peaks in the areas at the end of the proposed processing stream (BA 40, 10 & 11), where we typically observed an initial decrease in power after stimulus presentation followed by a low-amplitude power increase. Due to low amplitude of this late response, the latency estimate had higher variation and was more prone to errors related to low signal-to-noise ratio. Figure R2 reveals that in those cases peaks were often detected even before the word presentation (time 0), suggesting that there were multiple peaks in the early pre-stimulus and in the late post-stimulus periods. We preferred to allow our detection algorithm to choose the global maximum peak and disregard any other local maxima, rather than manually choose the 'proper' response peaks with visual inspection or restricting the time range.

One way of resolving this problem would be to exclude the last three regions from our analysis. Again, we prefer not to tamper with an automatic procedure for selecting active electrodes and the activated brain regions unless necessary. Figure 3a shows that these last three regions actually fit very well into a continuum of the sequence of induced brain responses in the proposed processing stream. We were therefore compelled to keep these regions in the analysis and show the limits of estimating the peak response in: the low amplitude in Figure 3a-b, large error bars of latencies in Figure 3b, and no classified group in Figure 3c. The third paragraph of Results has now been updated with the following statement:

The amplitude of these responses was gradually decreasing along the propagation stream and had the lowest values with the poorest estimates of the peak latency in the last three brain regions of Brodmann areas 40, 10 and 11 (Fig. 3b), where inconsistent peaks occurring at different latencies were observed.

2. boxplots and summary plots should be more informative: Please show individual data points, medians, whiskers or raincloud plots. Anything that shows the actual distribution and not just mean and SEM would help. I think this is crucial for the latency analysis, but applies to all other plots, too.

Continued from the previous response, we found the new box plots suggested by the Reviewer to confirm increased variance in peak latency observed in the last three brain regions of the proposed processing stream (Figure R2 below). This is why the error bars in Figure 3b for these regions were relatively larger compared to the other Brodmann areas. Peak power estimates of the response amplitude did not show such an increase in variance, which was higher in the early visual areas of the occipital cortex though not as high as the latency estimates for the last three brain regions (Figure R2). This observation was expected from the error bars in the original Figure 3b. However, the suggested new plots also reveal other features of the data point distributions that could not be seen in the original bar plots and are now clear in the revised Figure 3b. We agree with the Reviewer that the box plots are necessary to show additional information about the distributions, which does not compromise the clarity and interpretation of this figure. The general trends in the data across the studied brain regions are still very clear with an added benefit of distribution features within each brain region. We added the following sentence to the last paragraph of Methods and updated the figure legend accordingly:

Box plots were used to compare the medians, interquartile interval, range and outliers of data point distributions for the latency and power at peak maximum of the high gamma response to word presentations (Fig. 3b).

Similar or the other suggested data distribution plots would not benefit the remaining figures in the manuscript, in our opinion. Clarity and interpretation of the results presented in other figures would be compromised if we showed these additional features about data point distributions.

Figure R2. Comparison of bar and box plots for Figure 3b latency and peak power data. Bar plots from the original version of the figure (top row) are presented together with the corresponding new version of box plots (bottom row). Notice that the very large range of the latency data for BA 40, 10 and 11 is due to very small peak power values falling to low levels of signal-to-noise ratio for response detection.

3. The authors might want to cite Rich and Wallis (2017) Nat Comm and Watson (2017) EJN as resources that link HFA to spiking activity.

We were not aware of these new publications in the field and have now revised the following sentence in the last paragraph of the Introduction:

They comprise oscillatory and other asynchronous activities (Kucewicz et al., 2017), which are temporally coupled with firing discharges of neuronal populations (Rich and Wallis 2017; Watson et al., 2017).

And in the second paragraph of Discussion:

Most recent study in non-human primates confirmed the hierarchical organization and analogous spatiotemporal progression of neuronal spiking activity during short-term memory processing (Dotson et al., 2018). Another recent study showed a close relationship between neuronal spiking and field potential activity in the high gamma band (Rich and Wallis 2017), concluding that the high gamma activity is a useful biomarker of large-scale information processing. Our results with ECoG recordings of high gamma activity corroborate current evidence from the brain imaging studies for the visual processing stream (Milner and Goodale, 2006) and now provide supreme spatiotemporal resolution of the high gamma biomarker and the newly mapped memory effect to study the underlying neurophysiology.

We are grateful for recommending these helpful references, which are directly relevant to our on-going and future work.

4. It should be clearly noted when the author pooled data into a 'pseudo-population'. I think some analysis choices are rather unusual and subjective.

Given the small number of active electrodes in any one patient (mean of 6.85%) we had to pool active electrodes from different patients into a 'pseudo-population' to compare brain responses in specific brain regions. Vast majority of those electrodes showed a positive response to stimulus presentation as summarized in our previous revision response to a comment from Reviewer 1 (see Table R1 below). Apart from this initial screening step, all of the procedures for active electrode selection were fully automatic based on the standard deviation of trial-averaged power response. We are currently working on an improved method to automatically identify active electrodes in these recordings without any manual procedures (manuscript in submission to the Journal of Neural Engineering).

Another way of addressing this problem is to use high-density multi-channel recordings from arrays of meso- or micro-scale electrodes implanted in specific brain regions of a given patient. This would be expected to increase the number of active electrodes to be pooled into a proper population sampled from the same subject and allow a more appropriate within-subject analysis approach. Due to the technological limitations in this study we are not able to address this concern any further other than clearly stating how the populations of electrodes were pooled in the sixth paragraph of Methods:

Due to a small number of electrodes implanted in any one Brodmann area of a single patient, the active electrodes from specific Brodmann areas were pooled from all patients into pseudo-populations to compare brain responses in the identified brain regions.

We also now acknowledge that the electrodes were pooled from different patients in the first and the second paragraph of Results (pasted respectively below) and in the corresponding Figure 1-2 legends.

In order to obtain robust patterns of the high gamma responses, we identified 16 Brodmann area regions that showed consistent active electrode responses in multiple electrodes pooled from all patients into pseudo-populations that were used in all subsequent analyses (Fig.1b,c).

We summarized these differences for all active electrodes pooled from each of the identified Brodmann area regions from all patients and found a common 'flip-over' pattern of SME dynamics in all of the brain regions. The pooled electrode populations showed that SME first had negative polarity before word presentation for encoding (i.e. there was less high gamma power on the recalled word trials) followed by a flip to positive SME during word encoding (i.e. more high gamma power on the recalled word trials) and then finally flipping back to negative SME after word presentation (Fig. 2).

Table R1. Total number of the active electrodes showing increased (positive) and decreased (negative) power responses to stimulus presentation.
